# An automated resource for enhanced differential analysis

**DOI:** 10.1186/1471-2105-16-S15-P20

**Published:** 2015-10-23

**Authors:** Kai Wang, Charles A Phillips, Arnold M Saxton, Michael A Langston

**Affiliations:** 1 Department of Electrical Engineering and Computer Science, University of Tennessee, Knoxville, TN 37996-2250, USA; 2 Department of Animal Science, University of Tennessee Institute of Agriculture, Knoxville, TN 37996-4574, USA

## Background

Differential Shannon entropy (DSE) and differential coefficient of variation (DCV) have proven to be effective complements to differential expression (DE) in the analysis of gene co-expression data[1]. Because DSE and DCV measure difference in variability, rather than mere difference in magnitude, they can often identify significant changes in gene activity not reflected in mere mean expression level.

## Materials and methods

Thus, we have devised a general purpose, easy-to-use R package to calculate DSE and DCV. Dubbed *Entropy Explorer*, this package operates on two numeric matrices with identically labeled rows, such as case/control transcriptomic data. All functionality has been wrapped into one routine. With a single procedure call a user may select a metric, whether to display that metric, its raw and adjusted p-value, or both, whether to sort by metric or raw or adjusted p-value, and how many of the most highly ranked results to display.
